# Efficacy of low-protein diet for diabetic nephropathy: a systematic review of randomized controlled trials

**DOI:** 10.1186/s12944-018-0791-8

**Published:** 2018-06-19

**Authors:** Huan-gao Zhu, Zhao-shun Jiang, Pi-yun Gong, Dong-mei Zhang, Zhi-wei Zou, Qian-Zhang, Hui-mei Ma, Zhen-gang Guo, Jun-yu Zhao, Jian-jun Dong, Lin-Liao

**Affiliations:** 1 Division of Endocrinology, Qilu Hospital of Shandong University, Jinan, Shandong China; 2 Division of Endocrinology, The Ninth Hospital of Xi An, Shan xi, China; 3 Division of Endocrinology, The General Hospital of Jinan Military Command, 25 Shifan Road, Jinan, 250031 Shandong China; 4 Division of Endocrinology, Shandong Provincial Qianfoshan Hospital, Jinan, Shandong China; 5 Internal medicine department, Guanxian hospital of traditional Chinese medicine, Liaocheng, Shandong China

**Keywords:** Diabetic nephropathy, Protein restriction, Low-protein diet

## Abstract

**Background:**

A low-protein diet (LPD) is believed to be beneficial in slowing the progression of kidney disease. It is reported that low protein diet can improve protein, sugar and lipid metabolism, and reduce the symptoms and complications of renal insufficiency. However, there has been controversial regarding the effects of protein restriction on diabetic nephropathy (DN).

**Objective:**

To investigate the efficacy of LPD on renal function in patients with type 1 or 2 DN by meta-analysis of randomized controlled trials (RCTs).

**Design:**

PubMed, MEDLINE, EMBASE and China National Knowledge Infrastructure databases were searched. Eleven randomized controlled trials met the inclusion criteria, of which 10 were English and 1 was Chinese. The primary outcome was a change in glomerular filtration rate (GFR). The secondary outcome was a change in proteinuria. Random-effects models were used to calculate the standardized mean difference (SMD) and the corresponding 95% confidence intervals (CI). Subgroup analyses were also performed.

**Results:**

Our research indicated that LPD was not associated with a significant improvement in GFR (1.59 ml · min^−1^ · 1.73 m^−2^, 95% CI -0.57, 3.75, I^2^ = 76%; *p* = 0.15). This effect was consistent across the subgroups regardless of type of diabetes, course of diabetes and intervention period. Our results also showed that there was no significant difference on improvement of proteinuria in patients of LPD and those in normal-protein diet groups (− 0.48, 95%CI-1.70, 0.74, I^2^ = 94%, *p* = 0.44). Subgroup analysis revealed that LPD resulted in increased excretion of proteinuria in patients with type 2 diabetes (1.32, 95% CI 0.17, 2.47, I^2^ = 86%, *p* = 0.02).

**Conclusion:**

The present research showed that LPD was not significantly associated with improvement of renal function in patients with either type 1 or 2 diabetic nephropathy. Although these results do not completely eliminate the possibility that LPD is beneficial for patients with diabetic nephropathy, it does not seem to be significant benefit to renal function.

## Background

Diabetic nephropathy, develops in nearly half of patients with diabetes, is the leading cause of end-stage kidney disease (ESRD) worldwide, and is also substantially associated with increased risk of cardiovascular mortality [1, 2]. Multifactorial management have been proposed for diabetic nephropathy, such as diet therapy and well control of blood glucose, blood pressure and dyslipidemia. Among these, diet therapy has been suggested as the mainstay in the treatment of diabetic nephropathy. To delay the progression of ESRD in patients with diabetes, low-protein diet (LPD) is recommended by the American Diabetes Association guidelines to delay the progression of ESRD in patients with diabetes in 2008 [3–5].Since then, numerous studies focusing on the the efficacy of LPD for diabetic nephropathy have been performed. However, the results remains controversial [6–12]. Some studies reported the beneficial effects of LPD, which significantly slowed the increase in urinary albumin concentration or declined the glomerular filtration rate (GFR) or creatinine filtration rate (CCR) [13, 14]. While several researches revealed the opposite [15, 16]. Therefore, we conducted a systematic review and meta-analysis of randomized controlled trials (RCTs) to explore the effect of LPD on the progression of renal dysfunction and albuminuria in type 1 or type 2 diabetic patients with overt nephropathy.

## Methods

### Search strategy and inclusion criteria

We searched RCTs via PubMed, Medline, Embase and China National Knowledge Infrastructure databases, ClinicalTrials.gov from inception to December 2016 to identify relevant citations. The key words of the first step were “protein-restricted, diet” OR “diet, protein-restricted” OR “low protein diet” AND “diabetic nephropathies”. From these searches, studies evaluating the effects of LPD compared with control diet among diabetic patients were identified. Eleven studies met the inclusion criteria for our systematic review: 1.published in full text 2.use of a randomized control group 3.availability of outcome data for changes in GFR or CCR, and albuminuria or proteinuria in patients with type 1 or 2 diabetic nephropathy 4.RCTs of crossover design were excluded. Of the11 studies, 10 included trials were published in English-language medical journals.

### Data extraction

We extracted data related to the year of publication, patients and participants’ characteristics (age, sex, type and duration of diabetes or diabetic nephropathy), and outcomes (GFR, CCR or evaluated GFR, proteinuria or similar index, and serum albumin concentration). Also, we extracted data for patients’ compliance by integrating the data on actual protein intake evaluated for each study. We utilized these results to appraise the study quality and subsequent subgroup analyses were performed. The primary outcome was a change in GFR or CCr from baseline till the end of the diet intervention. The secondary outcome was the extraction of change in proteinuria.

### Statistical analysis

Data were combined by means of a random-effects model. The SD (standard deviation) were imputed by using interquartile ranges and full ranges. The methods of calculating the change-from-baseline SD are referenced in the Cochrane Handbook [17]. The standardized mean difference (SMD), which is calculated by dividing the mean values by the SD and which can be used to compare studies that report continuous outcomes by using different scales, was used to pool results from all studies that reported untransformed changes in urinary protein excretion.

### Risk of bias assessment

Risk of bias of included studies was estimated using the Cochrane Collaboration’s “risk of bias” assessment tool [18]. We assessed seven aspects: (1) blinding of participants, (2) allocation concealment, (3) sequence generation, (4) blinding of outcome assessment, (5) selective outcome reporting, (6) incomplete outcome data, (7) other bias by patients’ diet compliance. Since this study aimed to investigate the clinical effect of dietary intervention which encourages patients’ lifestyle modification, we considered that patients’ diet compliance was the most critical factor to generate risk of bias.

## Results

### Search results

**Fig. 1:**
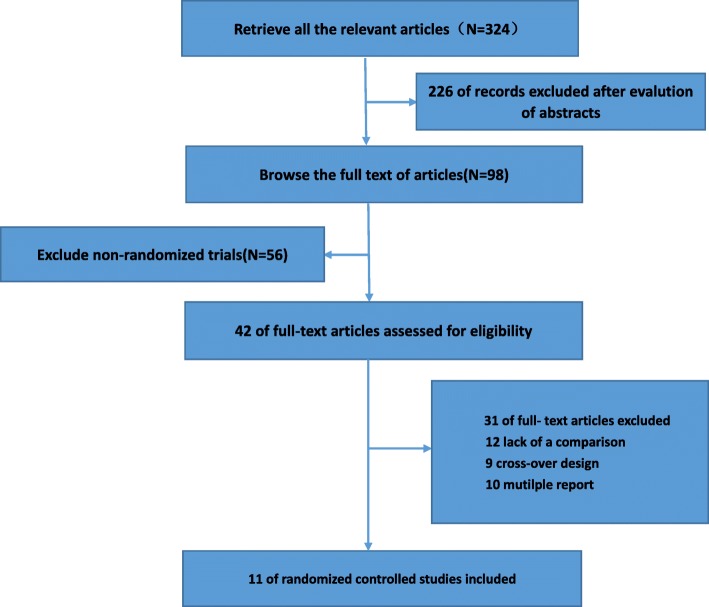
Flow diagram of the process for study selection

As shown in Fig. 1, we initially acquired 324 records through electrical database search. Of these, two hundred and twenty-six studies were excluded after evaluation of abstracts. Fifty-six non-random studies were excluded; and we selected 42 full text articles for detailed assessment for eligibility. Among these, we excluded thirty-one studies: twelve studies owing to lack of comparison, nine studies due to crossover design, ten studies were mutilple reports. Finally, we included 11 RCTs reporting the effects of LPD in diabetic patients.

### Characteristics of included studies

The included studies evaluated the effects of LPD in 687 diabetic patients. Study patients were middle-aged men and women, mostly obese or overweight (Tables 1 and 2). Five studies included patients with Type 1 diabetes, whereas six studies included patients with type 2 diabetes. Two studies included patients with both type 1 and type 2 diabetes patients and provided no separate information. Intervention period ranged from 2 to 60 months. However, allocation concealment was unclear in about half of the studies (Table 3**)**. Although the outcome assessment was not blinded to the assessors in any of the studies, the risk of bias is considered to be small since the outcome is objective.

**Table 1:** Characteristics of included studies

									LPD				NPD	
Author	Published year	Language	Male (%)	Mean Age (y)	Type of diabetes	Course of DM (y)	NO.	Protein intake (g.kg^−1^. d^−1^)	Proteinuria	GFR^a^ (mL. min^− 1^. 1.73 m^−2^)	NO.	Protein intake (g · kg^−1^ · d^− 1^)	proteinuria	GFR^a^ (mL. min^−1^ · 1.73 m^−2^)
Ciavarella A.	1987	English	56	37.1	T1DM	17.7	7	0.71	434 ± 244^b^	97 ± 34	9	1.44	452±200^b^	103 ± 28
Dullaart RP	1993	English	90	40.8	T1DM	23	14	0.6	36 (16, 83)^b, c^	131 ± 34	16	1.09	31 (19, 54)^b, c^	122 ± 26
Raal FJ	1994	English	36	30	T1DM	20	11	0.8	884 (87,9110)^b, d^	50 ± 19	11	2.0	1167 (80,4180)^b, d^	66 ± 28
Pijls L	1999	English	61	64	T2DM	6.8	58	0.8	21.4 (10, 40)^b, c^	81 ± 19	63	1.12	21.3 (8463.4)^b, c^	85 ± 24
HENRIK P.	1999	English	62	45	T1DM	29	14	0.6	397 (14,4091^d^	94	15	1.1	438(94,2934)^b, d^	92
Pijls LTJ	2002	English	65	63	T2DM	6.7	38	0.8	NA	69 ± 30	34	1.02	721 (502,1036)^b, d^	67 ± 32
Hansen HP	2002	English	63	40	T1DM	27	63	0.6	690(547,871)^b, e^	82 ± 19	68	1.14	NA	85 ± 23
Meloni C,	2004	English	55	43	T1DMorT2DM	22	40	0.8	2.4 ± 1.1^f^	43.7 ± 4.7	40	1.24	2.6 ± 0.8^e^	45 ± 5.1
Dussol B,	2005	English	83	52	T1DMorT2DM	15	22	0.8	NA	82 ± 21	25	1.2	NA	89 ± 27
D. Koya & M	2009	English	59	57	T2DM	NA	56	0.8	1.1 (0.4–3.2)^g^	63.5 ± 26.9	56	1.2	1.2 (0.5–2.9)^g^	61.1 ± 23.7
HY QIU	2012	Chinese	NA	62	T2DM	10	12	0.6	4.7 ± 2.12^e^	31.1 ± 10.41	11	0.8	3.96 ± 3.31^e^	36.75 ± 13.25

^a^Median (range), *DM* diabetes mellitus, *GFR* glomerular filtration rate, *T1DM* type 1 DM, *T2DM* type 2 DM, *LPD* low-protein diet, *NPD* normal-protein diet

^b^Measured as mg/24 h

^c^‾x; 95% CI in parentheses

^d^‾x; range in parentheses

^e^Geometric ‾x; 95% CI in parentheses

^f^Measured as g/24 h

^g^Measured as g/d

**Table 2:** Characteristics of included studies

Author (year)	NO.	BMI (kg/m^2^)	HbA1c (%)	Intervention period (months)
Ciavarella A.1987 [35]	16	NA	7.3	12
Dullaart RP.1993 [36]	30	24.1	7.8	24
Raal FJ.1994 [37]	31	24.9	13.0	6
Pijls L 1999 [38]	121	27.7	7.7	12
HENRIK P. 1999 [39]	29	25	8.5	2
Pijls LTJ 2002 [40]	72	27.8	7.7	28
Hansen HP 2002 [13]	131	25	9.8	48
Meloni C 2004 [41]	80	33.5	7.0	3
Dussol B 2005 [42]	47	NA	8.1	24
D. Koya & M 2009 [43]	112	24.6	7.6	60
Hong yu QIU 2012 [44]	23	NA	6.3	12

*BMI* body mass index, *HbA1c* haemoglobin A1C

**Table 3:** Risk of bias assessment

Study	Blinding of participants	Allocation concealment	Sequence generation	Blinding of outcome assessment	Selective outcome reporting	Incomplete outcome data	Other bias by patients’ diet compliance
Ciavarella A.	Y	Y	Y	Unclear	Unclear	N	N
Dullaart RP	Y	Unclear	Y	Unclear	Unclear	N	N
Raal FJ	Y	Y	Y	Unclear	Unclear	N	N
Pijls L	Y	Unclear	Y	Unclear	Unclear	N	N
HENRIK P.	Y	Y	Y	Unclear	Unclear	N	N
Pijls LTJ	Y	Unclear	Y	Unclear	Unclear	Y	N
Hansen HP	Y	Y	Y	Unclear	Unclear	N	N
Meloni C,	Y	Y	Y	Unclear	Unclear	Y	N
Dussol B,	Y	Unclear	Y	Unclear	Unclear	Y	N
D. Koya & M	Y	Y	Y	Unclear	Unclear	Y	N
Hong yu QIU	Y	Unclear	Y	Unclear	Unclear	Y	N

*Y* yes, *N* no

### Effects of low-protein diet on kidney function

**Fig. 2:**
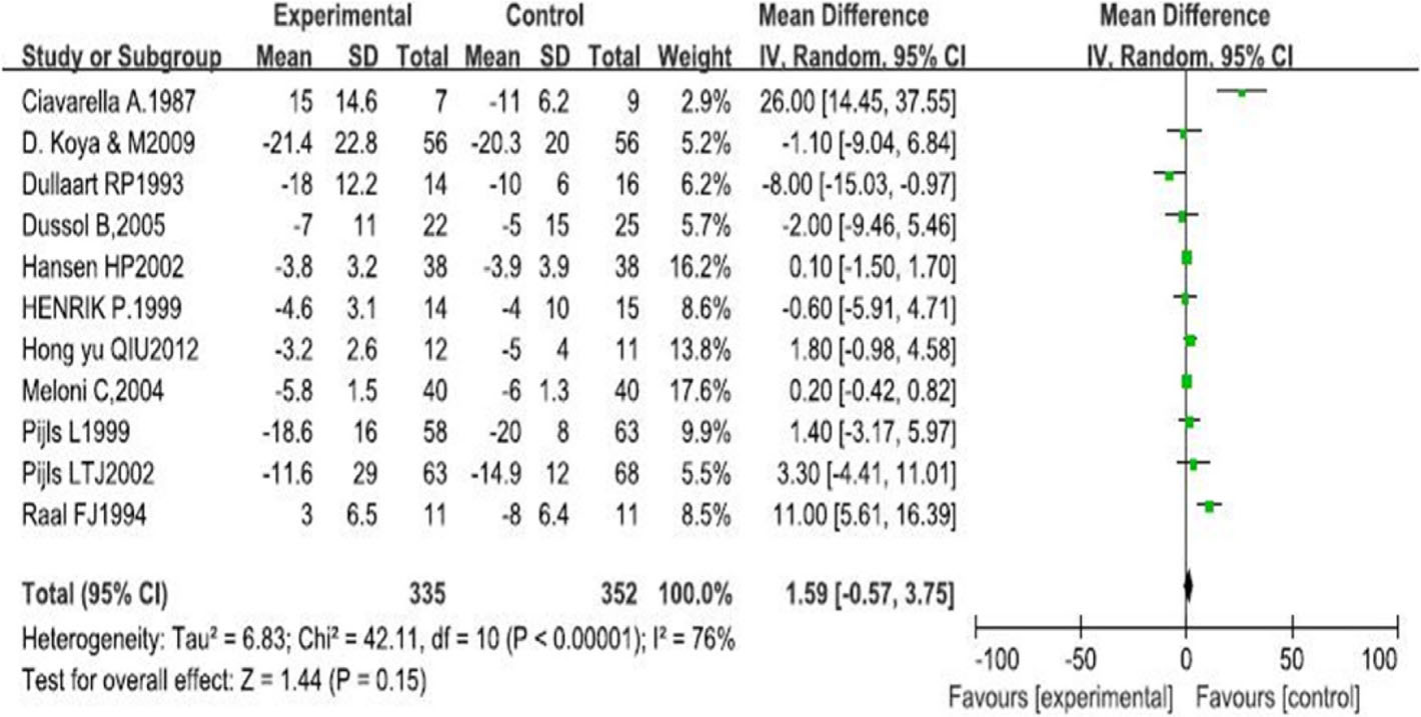
The forest plot of meta-analysis

**Fig. 3:**
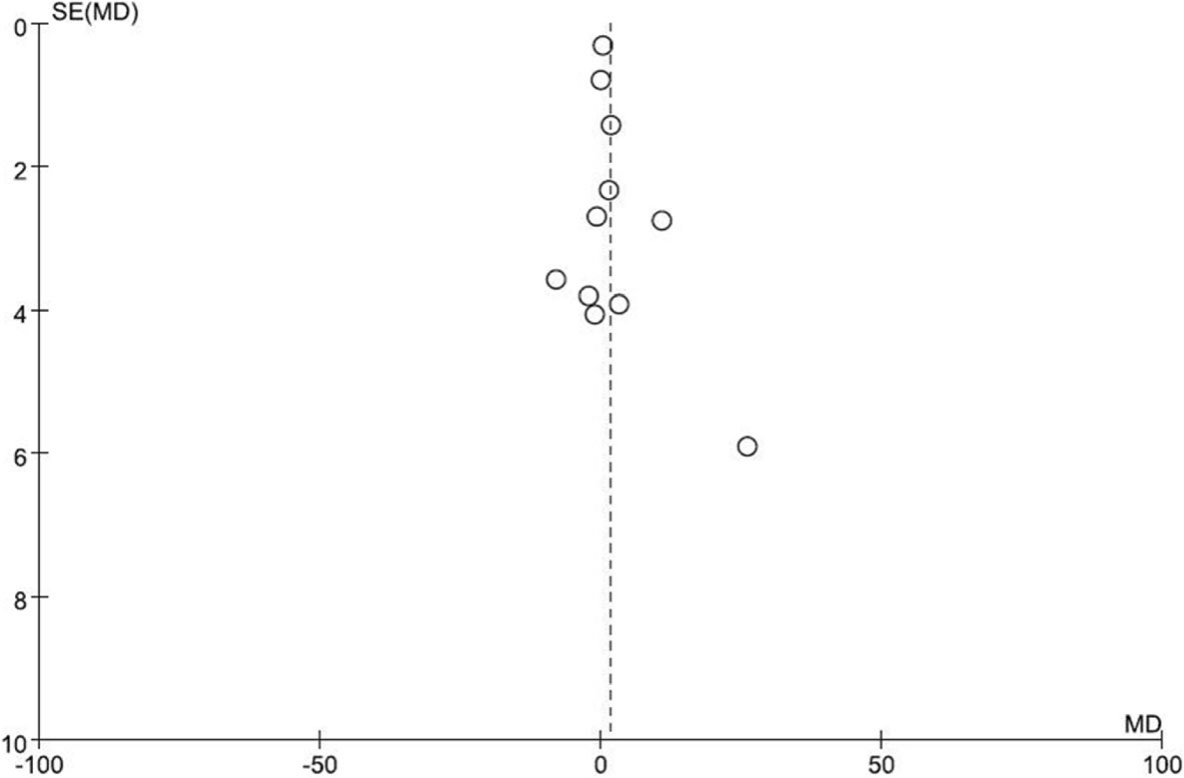
The funnel plot

There were no significant changes observed in GFR with the effects of LPD (95% CI-0.57, 3.75; *P* = 0.15, Fig. 2). We found significantly evident heterogeneity across the studies (I^2^ = 76%, *p* < 0.00001). However, the funnel plot showed no major asymmetricity (Fig. 3.)

### Effects of LPD on proteinuria or albuminuria

**Fig. 4:**
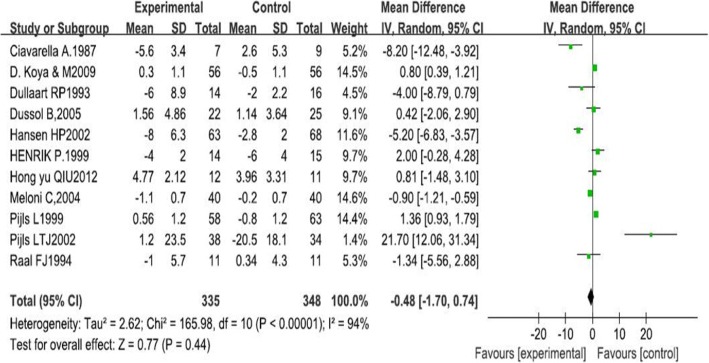
The forest plot of meta-analysis

Five different measurements of protein excretion were described in the trials: albumin excretion rate (mg/24 h), microalbuminuria (g/d), urine albumin excretion (UAE) (mg/24 h), 24 h proteinuria (g/24 h), and albuminuria (mg/24 h). Therefore, the SMD was used to compare these diverse measures. The standard mean difference showed no significant change in proteinuria after LPD (− 0.48, 95% CI − 1.70 to 0.74; *p* = 0.44; Fig. 4).

### Subgroup analyses

**Fig. 5:**
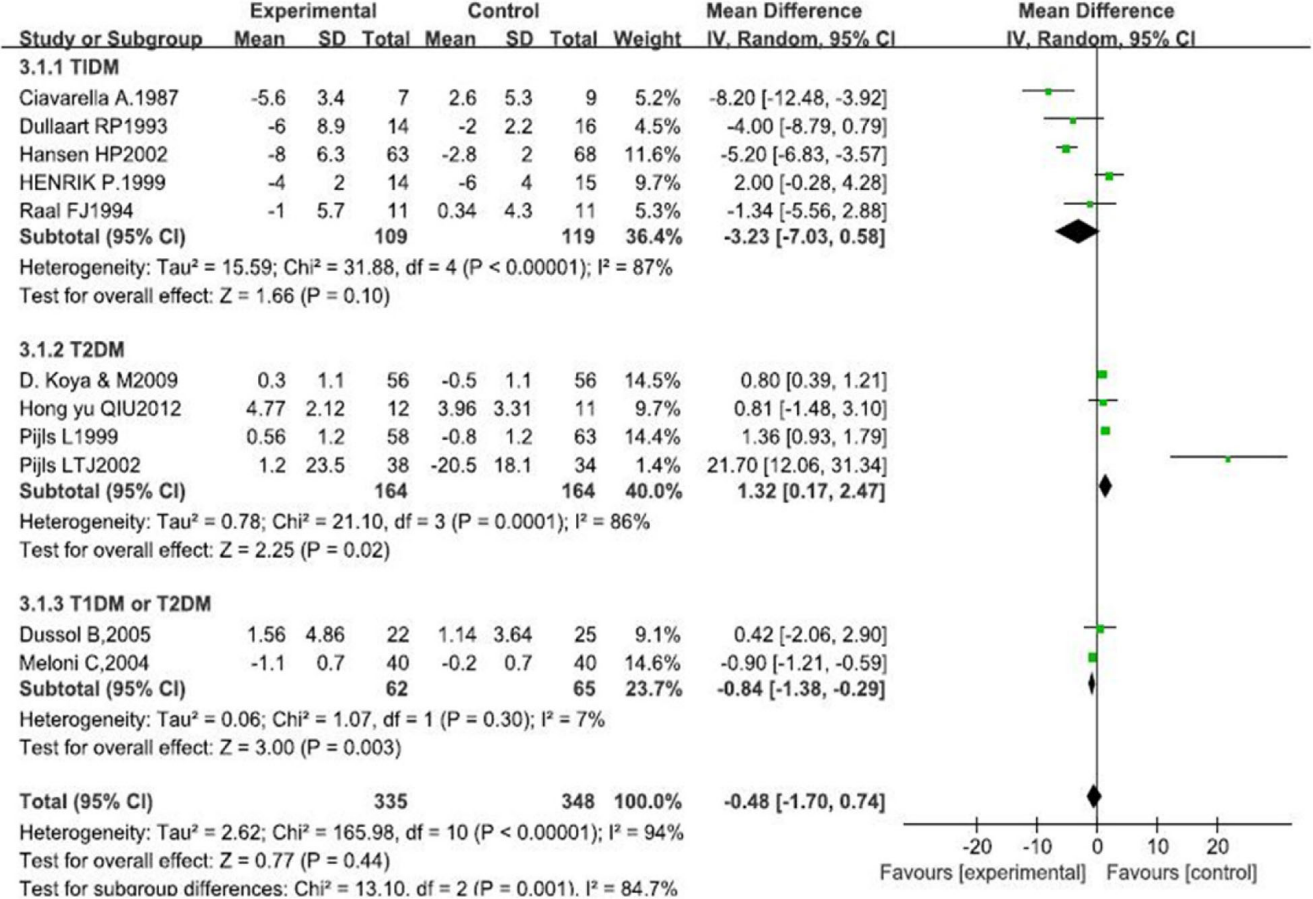
The forest plot of meta-analysis

Subgroup analysis was performed according to the clinical characteristics and study quality (Table 4). There were no significant differences observed in the changes in GFR between the subgroups based on the course of diabetes (1.76, 95% CI-0.5, 4.02, *p* = 0.98), type of diabetes (1.59, 95% CI-0.57, 3.75, *p* = 0.15), BMI (1.02, 95% CI-1.23, 3.28, *p* = 0.37) and intervention period (0.18, 95% CI-1.36, 1.72, *p* = 0.82). Changes in proteinuria demonstrated significant difference in the subgroups of type 2 diabetes (1.32, 95% 0.17, 2.47, *p* = 0.02; Fig. 5**)**. There was no significant difference observed between the subgroups of intervention period (− 0.20, 95%-1.43, 1.02, *p* = 0.24), course of diabetes (− 0.48, 95% CI-1.70, 0.74, *p* = 0.05) and BMI (− 0.13, 95% CI 1.62, 1.35, *p* = 0.66).

**Table 4:** Subgroup analyses for clinical characteristics and study quality

	GFR			Proteinuria		
Subgroups	Mean difference (95% CI)	I^2^ (%)	*p* Value	Mean difference (95% CI)	I^2^ (%)	*p* Value
Type of diabetes						
T1DM	4.46 (−2.59, 11.51)	90	0.21	−3.23 (−7.03, 0.58)	87	0.1
T2DM	1.61 (−0.57, 3.79)	0	0.15	1.32 (0.17, 2.47)	86	0.02
Mixed	0.19 (− 0.43,0.80)	0	0.55	−0.84 (−1.38,-0.29)	7	0.003
BMI						
BMI≤25	0.82 (− 11.12, 12.76)	89	0.89	−0.73 (−3.52, 2.06)	58	0.61
BMI > 25	0.22 (−0.35, 0.79)	0	0.44	0.52 (−1.82, 2.86)	98	0.66
Intervention period						
< 12 months	3.23 (−2.96, 9.42)	87	0.31	−0.04 (− 2.14, 2.06)	68	0.97
12-24 months	2.44 (−3.94, 8.82)	84	0.45	−1.17 (− 3.70, 1.36)	83	0.37
> 24 months	0.18 (−1.36, 1.72)	0	0.82	4.19 (−2.75, 11.12)	97	0.24
Course of DM						
≤ 10 years	1.83 (−0.44, 4.10)	0	0.11	3.93 (−0.33, 8.19)	88	0.07
> 10 years	1.87 (−1.19, 4.93)	85	0.23	−2.14 (−4.29, 0.01)	87	0.05

*BMI* body mass index, *GFR* glomerular filtration rate, *T1DM* type 1 diabetes mellitus, *T2DM* type 2 diabetes mellitus

## Discussion

In the present research, we included 11 RCTs that investigated the efficacy of LPD intervention in patients with type 1 or 2 diabetic nephropathy. Overall results of this meta-analysis indicated that, when compared with normal-protein diet, intake of LPD demonstrated no protective effect on diabetic nephropathy neither on improving GFR (1.59 ml · min^− 1^ · 1.73 m^−2^, 95% CI -0.57, 3.75, I^2^ = 76%; *p* = 0.15) nor proteinuria (− 0.48, 95% CI-1.70, 0.74, I^2^ = 94%, *p* = 0.44).

Diabetic nephropathy is the leading cause of progressive kidney disease, as the end stage renal disease consumed huge sum of money every year. Therefore, prevention of diabetic nephropathy is a major public health challenge. LPD is recommended by several guidelines as a basic measure for the treatment of diabetic nephropathy. However, the effect of LPD on diabetic nephropathy still remained controversial, and the results of our present study demonstrated a great meaning.

Previously, a number of studies have explored the role of LPD in patients with diabetic nephropathy. Several studies [19, 20] have evaluated the effects of LPD in animal models and indicated that LPD is renoprotective effects in renal diseases, even in advanced diabetic nephropathy via restoring autophagy through the suppression of the mechanistic target of rapamycin complex 1 (mTORC1) pathway in type 2 diabetes animal model [21]. One possible explanation is related to the amount of protein intake. Protein overload increases the secretion of glucagon from the pancreas, and the glucagon in turn induces direct dilatation of afferent arterioles in the glomeruli and subsequently increases intraglomerular pressure [22]. Protein overload also increases the secretion of insulin-like growth factor-1 (IGF-1) from the liver [23], and IGF-1 acts as a potent vasodilator of the renal vessels [24, 25]. Another possible explanation for the positive effect of LPD in diabetic nephropathy is linked to the renin-angiotensinsystem (RAS). Protein overload activates RAS, whereas LPD inhibits the intrarenal RAS [26–28]. Some studies demonstrated that blockade of RAS with either Angiotensin-Converting Enzyme Inhibitors (ACE-Is) or angiotensin receptor blockers (ARBs) slows down (but does not completely stop) the progression of diabetic nephropathy. The study published in BMJ Open concluded that a LPD was associated with a significant improvement in GFR (5.82 ml/min/1.73 m2, 95% CI 2.30 to 9.33, I2 = 92%; *n* = 624).While this study indicated that LPD was not associated with a significant improvement in GFR (1.59 ml · min^− 1^ · 1.73 m2, 95% CI -0.57, 3.75, I2 = 76%; *p* = 0.15). In the study we included, there was an article (Chinese article) that did not support the benefits of a low-protein diet, and the study published in BMJ Open did not retrieve this article. When we analyzed all the data included in the study, the weight of the data in this article is 13.8% (Fig. 1), which is a relatively high proportion. So we thought that the data from this study led to the final synthesis.

Unfortunately, a few clinical trials have reported disappointing results. A large-scale observational study that included 6213 individuals with type 2 diabetes found no clear benefits on renal parameters from LPD [29].The reason for these inconsistencies of the LPD benefits in diabetic nephropathy worth been further explored. As we all know, in clinical practice, the estimated GFR (eGFR) is the common indicator for the assessment of kidney function [30, 31]. But the measurement of creatinine to determine the eGFR has some limitations for the risk prediction, particularly in patients with reduced muscle mass [32]. Therefore, muscle loss can be misrepresented as an improvement in renal function. Additionally, dietary protein levels influence the blood sugar levels in both human and animal experiments [33]. These are the main confounding factors that affect the consistency in the outcomes of clinical studies.

In our research, crossover trials were excluded. Dr. Freeman pointed out that the crossover strategy is flawed and that it often gave rise to biased conclusions [34]. Given the fact that the crossover design may mask the effects of LPD on renal function, hence we excluded studies with crossover designs from the present study. Besides, the included number of patients was larger.

The significant benefits of LPD on renal diseases in animal and human studies did not impact the renoprotective strategies against diabetic nephropathy. On the basis of the available evidence in the literature and our study, there is no strong evidence for introducing a routine LPD as the standard nutritional intervention in diabetic nephropathy.

Our research has several advantages over previous studies. Firstly, our research includes Chinese and English databases, which are not available in the previously published study. Secondly, the most recent study was published 5 years ago. Our study incorporates the latest findings and is now a more comprehensive one. In addition, our study analyzed diabetes patients with different course of disease and different intervention time of low protein diet in detail.

Although the present analysis was based on RCTs, it has some limitations. There was considerable variation in the study subjects (type 1 or type 2 diabetic nephropathy), level of reduction of dietary protein, outcome analysis (GFR and proteinuria), and duration of study. These differences could explain some of the heterogeneity among the trials. Anyway, our research showed that LPD was not associated with a significant improvement of renal function in patients with both type 1 and type 2 diabetic nephropathy. Although these results do not completely eliminate the possibility that LPD is beneficial for patients with diabetic nephropathy, it does not seem to be significant benefit to renal function.

## Conclusions

In conclusion, the present research indicates that LPD has not conspicuously shown renoprotective effects in diabetic nephropathy. In future, we should merge our current knowledge of molecular genetics to reanalyze how an LPD works and determine the specific underlying molecular mechanisms. Meanwhile, large multicenter RCTs should be carried out to better understand the actual effect of an LPD on kidney outcomes in diabetic nephropathy.

## Abbreviations

ACE-Is: Angiotensin-Converting Enzyme Inhibitors
ARBs: angiotensin receptor blockers
CCR: creatinine filtration rate
CI: confidence intervals
DN: diabetic nephropathy
ESRD: end-stage kidney disease
GFR: glomerular filtration rate
LPD: low-protein diet
SD: standard deviation
SMD: standardized mean difference
